# The Role of Levosimendan in Patients with Decreased Left Ventricular Function Undergoing Cardiac Surgery

**DOI:** 10.3889/oamjms.2016.071

**Published:** 2016-06-28

**Authors:** Marija Bozhinovska, Gordana Taleska, Andrej Fabian, Maja Šoštarič

**Affiliations:** 1 *Clinical Department of Anesthesiology and Perioperative Intensive Therapy, Division of Cardiac Anesthesiology and Intensive Therapy, University Clinical Center Ljubljana, Zaloska 7, 1000 Ljubljana, Slovenia*; 2 *Institute of Physiology, Medical Faculty, University of Ljubljana, Slovenia*; 3 *Department of Vascular Neurology and Neurological Intensive Therapy, University Clinical Centre Ljubljana, Slovenia*

**Keywords:** Cardiac surgery, Levosimendan, Ventricular function, Heart failure, Myocardial dysfunction

## Abstract

The postoperative low cardiac output is one of the most important complications following cardiac surgery and is associated with increased morbidity and mortality. The condition requires inotropic support to achieve adequate hemodynamic status and tissue perfusion. While catecholamines are utilised as a standard therapy in cardiac surgery, their use is limited due to increased oxygen consumption. Levosimendan is calcium sensitising inodilatator expressing positive inotropic effect by binding with cardiac troponin C without increasing oxygen demand. Furthermore, the drug opens potassium ATP (KATP) channels in cardiac mitochondria and in the vascular muscle cells, showing cardioprotective and vasodilator properties, respectively. In the past decade, levosimendan demonstrated promising results in treating patients with reduced left ventricular function when administered in peri- or post- operative settings. In addition, pre-operative use of levosimendan in patients with severely reduced left ventricular ejection fraction may reduce the requirements for postoperative inotropic support, mechanical support, duration of intensive care unit stay as well as hospital stay and a decrease in post-operative mortality. However, larger studies are needed to clarify clinical advantages of levosimendan versus conventional inotropes.

## Introduction

Patients undergoing open heart surgery with cardiopulmonary bypass (CPB) experience global perioperative heart ischemia followed by reperfusion. This leads to different degrees of myocardial dysfunction due to free radical formation, impairment of the coronary vasculature and calcium overload [1, 2]. If severe enough this can cause postoperative low cardiac output syndrome (LCOS), a life-threatening complication with a prevalence of about 10% and a mortality of 17% [3].

Acute worsening of the left heart failure is commonly treated pharmacologically with intravenous positive inotropic agents, or mechanical support in severe cases [4]. Currently, beta-adrenergic agonists and phosphodiesterase inhibitors are most frequently used in clinical practice [4, 5]. Beta-adrenergic agents increase cardiac output (CO), cardiac index (CI) and stroke volume index by increasing intracellular cyclic adenosine monophosphate (cAMP) production and calcium influx to myocytes [6]. Phosphodiesterase inhibitors, like milrinone, do the same by inhibiting cAMP degradation [7]. This results in increased cellular energy demands and oxygen consumption, can trigger arrhythmias and can even be cardiotoxic [8, 9].

Levosimendan is a newer inotropic agent belonging to a class of calcium sensitizers that increase myocardial contractility by increasing the sensitivity of troponin C for calcium without increasing myocardial oxygen consumption [10]. Unlike some calcium sensitizers, levosimendan increases myocardial contractility without impairing diastolic cardiac function [11]. It displays vasodilator properties as well [12].

This review is focused on the impact of levosimendan in patients with left heart failure undergoing open heart surgery. Pharmacologic features of this drug will be briefly presented as well.

## Used Literature

The data sources used in this article are PubMed, Excerpta Medica, Reference Update, BIOSIS, Science Citation Index and Index Medicus. The abstract and papers were found by searching ([levosimendan ] and [surgery OR bypass OR valve OR CABG ]) were selected to the timing of the use of levosimendan and the type of intervention.

## Pharmacology of Levosimendan

Levosimendan is calcium sensitising inodilator that binds to troponin C, thereby enhancing myofilament responsiveness to calcium. This mechanism enhances myocardial contractility, maintaining the energy cost of contraction and intracellular concentration of calcium at a near normal level [1, 13]. Vasodilation is achieved by opening adenosine triphosphate (ATP) - sensitive KATP channels on smooth muscle cells of systemic and coronary vascular bed. Activation of KATP channels on cardiac mitochondria explains its cardioprotective effects [14].

Cardioprotective effects of levosimendan are short term (e. g. pre- and postconditioning, anti-ischemic, anti-stunning), and long term (e.g. anti-remodeling, anti-inflammatory and anti-apoptotic) [1]. Preconditioning refers to the ability of levosimendan to protect the heart from ischemia-reperfusion injury by preventing calcium overload, saving high energy phosphates and stabilising membrane potential due to increased potassium influx in cardiac mitochondria through activation of KATP channels [1, 14, 15].

In animal models, myocardial infarct size was significantly decreased after global ischemia- reperfusion in both levosimendan pretreatment (45 ± 2%) and ischemic preconditioning (38 ± 2%) compared with controls (52 ± 2%) (p < 0.05). Additionally, recent ex-vivo model study of cardiac ischemia-reperfusion showed that levosimendan pretreatment can decrease the infarct size and improve cardiac function [16].

The pharmacokinetics of levosimendan appears to be similar in patients with heart failure and healthy volunteers and remains relatively unaltered by gender, age and organ dysfunction [17]. Usually, a bolus dose of levosimendan of 6 to 24 µg/kg is used followed by continuous infusion of 24 h of 0.05-0.2 µg/kg in patients with heart failure NYHA classification II-IV [15, 18]. After a single dose or intravenous infusion, plasma concentration of levosimendan increases in a dose-dependent manner [4]. Approximately 97-98% is bound to plasma albumins, and its plasma half-life is one hour. Levosimendan is extensively metabolised through two pathways before excretion. The main pathway is conjugation into inactive metabolites, while the minor pathway is gut reduction into intermediate metabolite (OR-1855) and subsequent acetylation into an active metabolite (OR 1896) [4].

The OR-1896 exhibits similar haemodynamic influence as levosimendan and a half-life of approximately 80 hours [19]. This most likely explains the sustained improvements in ventricular performance and natriuretic peptide levels compare to traditional inotropes [20, 21]. The most often reported adverse effect is a headache, observed in 10 % of the healthy volunteers receiving levosimendan [4]. Other side effects that probably appear due to the vasodilatatory effects of levosimendan include dizziness, nausea and palpitations. Levosimendan acutely reduces systemic vascular resistance and ventricular filling pressure. Consequently, in some patients with heart failure, it lowers blood pressure and vasopressors are required in the first few hours or days of administration [22].

## Effects of Levosimendan in Patients with Heart Failure

Levosimendan has been used in the treatment of heart failure (HF) over the past decade. Studies have shown clinical improvement in terms of hemodynamic parameters, cardiac function, shorter overall in-hospital stay, while data for long-term mortality is still controversial [23].

In a Randomized study of safety and effectiveness of Levosimendan in patients with left ventricular failure after acute myocardial infarction (RUSSLAN), 504 patients were randomised to receive either infusion of levosimendan or placebo [24]. Patients in levosimendan group experienced reduced short-term and long term mortality at 14 days (11.7% vs. 19.6%, p = 0.031) and on 180 days (22.6% vs. 31.4%, p = 0.053) compared to placebo group [11,24].

In the randomised multicenter evaluation of intravenous Levosimendan efficacy (REVIVE II) study 600 patients with acute decompensated heart failure (ADFH), were treated with levosimendan (299 patients) or placebo (301 patients) infusion in addition to standard therapy. 58 patients in the levosimendan group and only 44 patients in the placebo group got improved at 6 h, 24 h and 5 days. By contrast, 82 patients in the placebo but only 58 patients in the levosimendan group got worse (p = 0.015) [10]. In respect of mortality at 90 days, there was no difference between the groups [25].

In SURVIVE study (Survival of patients with acute HF in need of intravenous inotropic support), which randomised 1327 patient in a double-blind fashion to either dobutamine or levosimendan, short-term (31 days) and long-term (180 days) mortality was evaluated in patients with severe ADHF [26]. Patients received bolus of levosimendan 12 µg/kg in 10 min, followed by 0.1-0.2 µg/kg/min continuous infusion for 24 h [10]. Mortality rate was evaluated at 5 days, 2 weeks, 1 and 6 months and the results showed reduced mortality for levosimendan group by 27%, 14%, 13%, and 6.4% respectively, compared to dobutamine group, however with no statistically significant difference [27].

## Levosimendan in Cardiac Surgery

Evidence shows the benefit of coronary surgery over medical treatment in patients suffering from both decreased left ventricular function and coronary artery disease [20, 28]. Patients undergoing cardiac surgery with high-risk profile require peri-operative and post-operative inotropic support and levosimedan seems to be an attractive option in such patients [23]. Different meta-analyses have suggested mortality benefits in patients undergoing cardiac surgery receiving levosimendan. Landoni and co-workers analysed 45 randomised clinical trials including 4580 patients and found reduced mortality with an OR of 0.35 (95% CI 0.18-0.71, p = 0.003) in patients treated with levosimedan [29]. Maharaj and co-workers analyzed 17 studies including 729 patients after coronary revascularization treated with levosimendan versus control, and found mortality benefit with an OR of 0.40 (95% confidence interval (CI) 0.21 to 0.76, *P* = 0.005) [28].

Harrison and co-workers analysed 14 randomised controlled trials including 1155 patients, evidence showed decreased mortality rate in patients intraoperatively treated with levosimendan (risk difference −4.2%, 95% CI −7.2% to −1.1%, p = 0.008), in patients with reduced ejection fraction (EF) these benefits were greatest [28].

Lim and co-workers analysed 14 studies of patients with reduced left ventricular ejection fraction (LVEF) undergoing cardiac surgery. Results showed reduced early mortality (5.5% vs. 9.1%), reduced Intensive care unit stay (ICU) 1.01 (1.61–0.42) days and reduced postoperative acute renal injury (ARI) (7.4% vs. 11.5%) in patients treated with levosimendan [31].

Reports from several clinical studies have compared levosimendan versus milrinone, placebo, dobutamine or intra-aortic balloon pump (IABP)[23]. Patients with preserved or reduced left ventricular function received levosimendan in pre, intra or post- operative setting (period).

## Pre-Operative Use of Levosimendan in Cardiac Surgery

In a trial by Levin and co-workers, 252 high-risk patients with severely reduced LVEF scheduled for coronary artery bypass grafting (CABG), were randomized to receive either levosimendan or placebo infusion 24 hours pre-operatively. Levosimendan was started with a loading dose of 10 µg/kg infusion for 1 h than followed by continuous infusion of 0.1 µg/kg/ min infusion for 23 h. Reduced mortality was seen in levosimendan group compared to placebo (3.9% vs. 12.8%; P = 0.05), decreased incidence of LCOS (7.1% vs. 20.8%; P = 0.05) and complicated weaning from CPB (2.4% vs. 9.6%; P = 0.05). In patients treated with levosimendan results showed lower requirement for other inotropes (7.9% vs. 58.4%; P = 0.05), vasopressors (14.2% vs. 45.6%; P = 0.05), and lower requirements for IABP (6.3% vs. 30.4%; P = 0.05) compared to placebo group [32].

In a randomised, double-blind, placebo-controlled study by Leppikangas and co-workers, 24 patients were enrolled and received either levosimendan or placebo infusion 24 hours before surgery. Results showed improved hemodynamic parameters in levosimendan group compared to placebo as well as higher levels of levosimendan’s metabolites compared to other studies that use levosimendan in intra-operative setting [33].

## Intra-operative Use of Levosimendan

Several trials assessed the effect of levosimendan started after induction of anaesthesia or before initiation of CPB in patients with preoperatively impaired LVEF. Although the difference in mortality rate on patients treated with levosimendan compared to placebo or other inotrops showed no statistical significance difference, secondary end points such as ICU stay, hospital stay, mechanical ventilation, requirements for mechanical support and other inotrops were significantly decreased [34, 35].

In the randomised double-blind trial, Triapepe and co-authors compared placebo versus levosimendan in 106 patients scheduled for elective CABG. Patients receive levosimendan slow i.v. (24 µg kg (-1)) or placebo over 10 min before initiation of CPB. Results showed significant reduction of the length of ICU stay and tracheal intubation time, lower level of postoperative troponin I (P < 0.0001) and higher cardiac index (P < 0.0001), without a statistically significant difference in the study groups in respect of 30-day mortality [36].

The effect of levosimendan on the postoperative outcome with respect to the time of administration (early versus late) was assessed in 159 patients with impaired LVEF less than 35%, in a trial conducted by Treskatsh and co-workers. Patients that received levosimendan intra-operatively up to the first hour after ICU admission showed better survival (49/70) compared to patients treated with levosimendan infusion after the first hour of ICU admission (41/89). The survivors in the late start group showed increased incidence of in-hospital mortality (p = 0.004) and increased 1-year mortality (p = 0.027). Additionally, the incidence of renal dysfunction (p = 0.002), requirements for renal replacement therapy (RRT) (p = 0.032) and duration of mechanical ventilation (p = 0.002) were significantly increased in the late start group [37].

Järvelä and co-workers randomized 24 patients with severe aortic stenosis and left ventricular hypertrophy scheduled for aortic valve replacement (AVR). Levosimendan group received bolus, followed by i.v. continuous infusion (0.2 µg/kg/min), and started after induction of anaesthesia. LVEF was maintained in levosimendan group while it decreased in the placebo group, without significant difference [34].

Benefits of Levosimendan compared to IABP were assessed in a prospective randomised trial of 90 patients with coronary artery disease and LVEF less than 35%, scheduled for surgery [35]. Patients in levosimendan group had significantly shorter ICU stay compared to other groups (*p* < 0.01); six hours after surgery level of cardiac troponin (cTnI) was increased in all groups but significantly lower in the levosimendan group. Concentration of cTnI at the first postoperative day was 4.67 ng/mL (1.04 to 8.20), 2.40 ng/mL (1.17 to 5.14), and 2.99 ng/mL (1.52 to 7.40) and levels decreased at second post-operative day. Furthermore, levosimendan improved hemodynamic parameters and contributed to lower cTnI levels compared to preoperative IABP [35].

Elahi et al. also evaluated the impact of preoperative use of either IABP or levosimendan on patients outcome, and data showed that in cardiac surgery patients with LCOS both improved cardiac function, although provided no evidence to suggest weather IABP or levosimendan is superior in treatment of patients with LCOS with regard to morbidity and mortality [38].

In a trial by Lahtinen et al. 207 patients scheduled for valve ± coronary surgery were randomised into placebo or levosimendan group and the infusion was initiated after induction of anaesthesia. Results showed reduced the number of patients with HF in levosimendan group (risk ratio 0.26, 95% CI0.16-0.43, p = 0.001) but they were more likely to require noradrenalin and less likely to require IABP compared to placebo group. No significant difference in in-hospital and six months mortality between the groups [39].

## The Use of Levosimandan in Post-Operative Course

Several studies have evaluated the effect of levosimendan on patients with LCOS (defined as CI < 2.2 l/min/m2, mixed venous saturation < 60%, pulmonary wedge pressure > 18 mmHg) after cardiac surgery. Levin and colleagues randomised 253 parents with LCOS after surgery, to receive either levosimandan or dobitamine. Patients in levosimendan group showed reduced requirements for inotrops, vasopressors, IABP use, as well as decrease postoperative mortality (7.1 vs. 15.9%) [23, 40].

Alvarez and colleagues randomised 41 patients after open heart surgery on CPB to either levosimendan or dobutamine administered in the treatment of post operative LCOS. Although both drugs are effective in LCOS treatment, short term hemodynamics was improved, levosimendan showed significantly greater effects in improving CI (2.9 (0.3) l/min/m2 vs. 2.4 (0.2) l/min/m2, p = 0.05) as well as significantly reduced systemic and pulmonary vascular resistances, also decreased pulmonary capillary wedge and central venous pressure(CVP) [41]. Al–Shawaf and colleagues conducted a randomised trial comparing levosimendan vs milrinone for the management of LCOS in diabetic patients after cardiac surgery with LVEF < 35%. Significantly higher mixed venous saturation, cardiac indices, lower pulmonary capillary wedge pressures and oxygen extraction rates were found In levosimendan group. Therefore, it was suggested that levosimendan was more effective in treating hemodynamic worsening of post operative LCOS [42].

## Treatment of Levosimandan in Patients with Right Heart Failure

Several trials have investigated the effects of levosimendan on patients with biventricular HF and cardiogenic shock related to acute myocardial infarction, and demonstrated reduced right ventricle (RV) afterload and increased RV contractility [1, 10]. Russ and colleagues evaluated the effect of levosimandan versus conventional therapy (dobutamine and norepinephrine) in patients with cardiogenic shock due to myocardial infarction treated with percutaneous coronary intervention (PCI). Results showed significant increase in cardiac power index in both left and right ventricle (2.1 ± 0.1 to 3.0 ± 0.2, p < 0.01) and (0.14 ± 0.19 to 0.18 ± 0.12, p < 0.001) respectively, and also decreased pulmonary vascular resistance [43]. In a study, Yulmas and colleagues randomised 40 patients with significant biventricular systolic dysfunction to receive either levosimendan or dobutamine and in levosimendan group doppler echocardiographic markers of systolic function and tricuspid annulus were significantly improved (15% ± 12% vs. 2% ± 6%, P < 0.001) versus dobutamine [44].

## Postoperative Kidney Function in Patients Treated with Levosimendan

Postoperative renal dysfunction in patients after cardiac surgery is an important complication and independently is associated with increased mortality, morbidity, prolonged ICU and prolonged overall in-hospital stay [45]. Several trials have investigated the effect of levosimendan in patients with LCOS after cardiac surgery the results showed that the drug can increase renal blood flow (RBF), CO and by this features its reno-protective effects were recognised [46, 47]. In one of the latest meta-analysis evaluating the renal effect of levosimendan continuous infusion 0.1 – 0.2 µg/kg/min after loading dose of 6-24 µg/kg for 24 h or only loading dose 24 µg/kg in 1h versus placebo and/or other inotropic drugs in 13 trials and 1345 patients, the authors found that levosimendan reduces the incidence of postoperative AKI, RRT, postoperative mortality, duration of mechanical ventilation and ICU stay. In a prospective, double-blind clinical trial Baysal and colleagues randomised 128 patients undergoing mitral valve surgery with LVEF less 45 % were to receive either levosimendan 6 µg/kg/min as a loading dose then 0.1 µg/kg/min after aortic clamp removal compared to standard inotropic therapy. Patients in the levosimendan group showed improved postoperative renal clearance and higher values of estimated glomerular filtration rate (eGFR) on day 1 and day 3 compared to control group p = 0.0001, p = 0.0009 respectively and the need of RRT was reduced [48].

In a retrospective observational study, Balzer and colleagues analysed 46 patients with reduced LVEF treated with levosimendan as a continuous infusion 0.1 µg/kg/min with respect to the timing of it’s administration during surgery early versus late start in ICU. Intra-operatively 61% of the patients received levosimendan versus 39% of patients treated with levosimendan in ICU and the results showed significantly reduced creatinine plasma levels p= 0.009, reduced incidence of postoperative renal dysfunction (67.9% vs. 94.4%, p = 0.033) and also reduced the duration of the RRT [49].

Knezevic and al. evaluate the reno-protective role of levosimendan versus standard inotropic therapy, in 94 patients included for heart transplantation. In the first week after transplantation, patients in levosimendan group showed increased eGFR (62% vs.12%, p = 0.002) with lower incidence of AKI (28% vs. 6%, p = 0.01). In the first tree months, 19% of patients with AKI died versus 8% of patients without AKI (p = 0.37). In the study population, the one-year survival rate was 87%, without a statistically significant difference in mortality rate between the groups (11% in levosimandan vs. 15% in control, p = 0.54)[50].

## Cost-Effectiveness of Treatment with Levosimendan

Economic and health analysis of treatment with levosimendan in acute decompensated heart failure showed that it is cost effective. However, in cardiac surgery, there is limited data regarding it’s cost effective. In an observational study with 292 patients with acute heart failure, Fedele and colleagues preformed cost-effectiveness analysis of treatment with levosimendan versus the standard treatment with dobutamine. It was found that per capita cost of treatment with levosimendan is higher in the initial hospitalisation, but when re- hospitalisations were considered costs, they were significantly lower [23, 51]. Therefore, available data imply that treatment with levosimendan in cardiac surgery positively affects the elements that are likely to increase the costs of treatment: shorter ICU stay, fewer requirements for IABP and renal replacement therapy [1]. Furthermore, improved indirect indicators may show the advantage of levosimendan in cost-effectiveness over dobutamine, particularly when administered in high-risk patients [23].

In conclusion, clinical studies show that levosimendan enhances the cardiac function and improves the hemodynamic, pulmonary and general condition in patients after cardiac surgery in both reduced and normal LVEF. Pre operative use of levosimendan in high-risk patients with severely reduced LVEF may reduce the requirements for inotropic support, mechanical support, duration of intensive care unit stay as well as hospital stay and decrease post operative mortality. The greatest benefits may be seen in high-risk patients where levosimandan is applied in preoperative setting 24 h before surgery.

## References

[1] Toller W, Heringlake M, Guarracino F, Algotsson L, Alvarez J, Argyriadou H (2015). Preoperative and perioperative use of levosimendan in cardiac surgery: European expert opinion. Int J Cardiol.

[2] Suleiman MS, Zacharowski K, Angelini GD (2008). Inflammatory response and cardioprotection during open-heart surgery: the importance of anaesthetics. Br J Pharmacol.

[3] Malliotakis P, Xenikakis T, Linardakis M, Hassoulas J (2007). Hemodynamic effects of Levosimednan for low cardiac output after cardiac surgery: A case series. Hellenic J Cardiol.

[4] Raja SG, Rayen BS (2006). Levosimendan in cardiac surgery: current best available evidence. Ann Thorac Surg.

[5] Amsallem E, Kasparian C, Haddour G, Boissel J P, Nony P (2005). Phosphodiesterase III inhibitors for heart failure. Cochrane Database of Syst. Rev.

[6] Overgaard CB, Dzavík V (2008). Inotropes and vasopressors: review of physiology and clinical use in cardiovascular disease. Circulation.

[7] Packer M (1993). The search for the ideal positive inotropic agent. N Engl J Med.

[8] Lee JC, Downing SE (1980). Cyclic AMP and the pathogenesis of myocardial injury. Res Commun Chem Pathol Pharmacol.

[9] Ferrick KJ, Fein SA, Ferrick AM (1990). Effect of milrinone on ventricular arrhythmias in congestive heart failure. Am J Cardiol.

[10] Nieminen MS, Fruhwald S, Heunks LM, Suominen PK, Gordon AC, Kivikko M (2013). Levosimendan: current data, clinical use and future development. Heart Lung Vessel.

[11] Antoniades C, Tousoulis D, Koumallos N, Marinou K, Stefanadis C (2007). Levosimendan: beyond its simple inotropic effect in heart failure. Pharmacol Ther.

[12] Caimmi PP, Kapetanakis EI, Beggino C, Molinari C, Giustini G, Crosio E (2011). Management of acute cardiac failure by intracoronary administration of levosimendan. J Cardiovasc Pharmacol.

[13] Kamath SR, Jaykumar I, Matha S (2009). Levosimendan. Indian Pediatr.

[14] Pathak A, Lebrin M, Vaccaro A, Senard JM, Despas F (2013). Pharmacology of levosimendan: inotropic, vasodilatory and cardioprotective effects. J Clin Pharm Ther.

[15] Kasikcioglu HA, Cam N (2006). A review of levosimendan in the treatment of heart failure. Vasc Health Risk Manag.

[16] Lepran I, Pollesello P, Vajda S, Varró A, Papp G J (2006). Preconditioning effects of levosimendan in a rabbit cardiac ischemia–reperfusion model. J. Cardiovasc. Pharmacol.

[17] Ng TM (2004). Levosimendan, a new calcium-sensitizing inotrope for heart failure. Pharmacotherapy.

[18] Jonsson N E, Antila S, McFadyen L, Lehtonen L, Karlsson M (2003). Population pharmacokinetics of levosimendan in patients with congestive heart failure. Br J Clin Pharmacy.

[19] Moreno N, Tavares-Silva M, Lourenço AP, Pinto J O, Henriques-Coelho T, Leite-Moreira AF (2014). Levosimendan: The current situation and new prospects. Rev Port Cardiol.

[20] Eriksson HI, Jalonen JR, Heikkinen LO, Kivikko M, Laine M, Leino KA (2009). Levosimendan facilitates weaning from cardiopulmonary bypass in patients undergoing coronary artery bypass grafting with impaired left ventricular function. Ann Thorac Sure.

[21] Kivikko M, Lehtonen L, Colucci WS (2003). Sustained hemodynamic effects of intravenous levosimendan. Circulation.

[22] Nieminen MS, Buerke M, Cohen-Solál A, Costa S, Édes I, Erlikh A, Franco F (2016). The role of levosimendan in acute heart failure complicating acute coronary syndrome: A review and expert consensus opinion. Int J Cardiol.

[23] Shi WY, Li S, Collins N, Cotten DB, Bastian BC, James AN, Mejia R (2015). Peri- operative Levosimendan in Patients Undergoing Cardiac Surgery: An Overview of the Evidence. Heart Lung Circ.

[24] De Luca L, Colucci WS, Nieminen MS, Massie BM, Gheorghiade M (2006). Evidence-based use of levosimendan in different clinical settings. Eur Heart J.

[25] Delaney A, Bradford C, McCaffrey J, Bagshaw SM, Lee R (2010). Levosimendan for the treatment of acute severe heart failure: A meta-analysis of randomised controlled trials. Int J Cardiol.

[26] Mebazaa A, Nieminen MS, Filippatos G S, Cleland JG, Salon JE, Thakkar R, Padley RJ, Huang B, Cohen-Solal A (2009). Levosimendan vs. dobutamine: outcomes for acute heart failure patients on beta-blockers in SURVIVE. Eur J Heart Fail.

[27] Cohen-Solal A, Logeart D, Huang B (2009). Lowered B type natriuretic peptide in response to levosi mendan or dobutamine treatment is associated with improved survival in patients with severe acutely decompensated heart failure. J Am Coll Cardiol.

[28] Harrison R W, Hasselblad V, Mehta RH, Levin R, Harrington RA, Alexander JH (2013). Effect of levosimendan on survival and adverse events after cardiac surgery: a meta-analysis. J Cardiothorac Vasc Anesth.

[29] Landoni G, Mizzi A, Biondi-Zoccai G (2010). Reducing mortality in cardiac surgery with levosimendan: a meta-analysis of randomized controlled trials. J Cardiothorac Vasc Anesth.

[30] Maharaj R, Metaxa V (2011). Levosimendan and mortality after coronary revascularisation: a meta-analysis of randomised controlled trials. Crit Care.

[31] Lim JY, Deo SV, Rababa’h A, Altarabsheh SE, Cho YH, Hang D (2015). Levosimendan reduces mortality in adults with left ventricular dysfunction undergoing cardiac surgery: a systematic review and meta-analysis. J Card Surg.

[32] Levin R, Degrange M, Del Mazo C, Tanus E, Porcile R (2012). Preoperative levosimendan decreases mortality and the development of low cardiac output in high-risk patients with severe left ventricular dysfunction undergoing coronary artery bypass grafting with cardiopulmonary bypass. Exp Clin Cardiol.

[33] Leppikangas H, Järvelä K, Sisto T, Maaranen P, Virtanen M, Lehto P (2011). Preoperative levosimendan infusion in combined aortic valve and coro- nary bypass surgery. Br J Anaesth.

[34] Järvelä K, Maaranen P, Sisto T, Ruokonen E (2008). Levosimendan in aortic valve surgery: cardiac performance and recovery. J Cardiothorac Vasc Anesth.

[35] Lomivorotov VV, Boboshko VA, Efremov SM (2012). Levosimendan versus an intra-aortic balloon pump in high-risk cardiac patients. J Cardiothorac Vasc Anesth.

[36] Tritapepe L, De Santis V, Vitale D, Guarracino F, Pellegrini F, Pietropaoli P (2009). Levosimendan pre-treatment improves outcomes in patients undergoing coronary artery bypass graft surgery. Br J Anaesth.

[37] Treskatsch S, Balzer F, Geyer T, Spies CD, Kastrup M, Grubitzsch H (2015). Early levosimendan administration is associated with decreased mortality after cardiac surgery. J Crit Care.

[38] Elahi MM, Lam J, Asopa S, Matata BM (2011). Levosimendan versus an intra-aortic balloon pump in adult cardiac surgery patients with low cardiac output. J Cardiothorac Vasc Anesth.

[39] Lahtinen P, Pitkänen O, Pölönen P (2011). Levosimendan reduces heart failure after cardiac surgery – a prospective, randomized, placebocontrolled trial. Crit Care Med.

[40] Levin R, Porcile R, Salvagio F, Del Mazo C, Botbol A, Tanus E (2009). Levosimendan reduces mortality in post-operative low cardiac output syndrome after coronary surgery. Circulation.

[41] Alvarez J, Bouzada M, Fernández AL (2006). Hemodynamic effects of levosimendan compared with dobutamine in patients with low cardiac output after cardiac surgery. Rev Esp Cardiol. (Engl. Ed.).

[42] Al-Shawaf E, Ayed A, Vislocky I, Radomir B, Dehrab N, Tarazi R (2006). Levosimendan or milrinone in the type 2 diabetic patient with low ejection fraction undergoing elective coronary artery surgery. J Cardiothorac Vasc Anesth.

[43] Russ MA, Prondzinsky R, Carter JM, Schlitt A, Ebelt H, Schmidt H, Lemm H (2009). Right ventricular function in myocardial infarction complicated by cardiogenic shock: Improvement with levosimendan. Crit Care Med. http://dx.doi.org/10.1097/CCM.0b013e3181b0314a.

[44] Yilmaz M B, Yontar C, Erdem A, Karadas F, Yalta K, Turgut O (2009). Comparative effects of levosimendan and dobutamine on right ventricular function in patients with biventricular heart failure. Heart Vessels.

[45] Niu ZZ, Wu SM, Sun WY, Hou WM, Chi YF (2014). Perioperative levosimendan therapy is associated with a lower incidence of acute kidney injury after cardiac surgery: a meta- analysis. J Cardiovasc Pharmacol.

[46] Zorlu A, Yucel H, Yontar OC, Karahan O, Tandogan I, Katrancioglu N (2012). Effect of levosimendan in patients with severe systolic heart failure and worsening renal function. Arq Bras Cardiol.

[47] Zhou C, Gong J, Chen D, Wang W, Liu M, Liu B (2016). Levosimendan for prevention of acute kidney injury after cardiac surgery: A Meta-analysis of controlled trials. Am J Kidney Dis.

[48] Baysal A, Yanartas M, Dogukan M, Gundogus N, Kocak T, Koksal C (2014). Levosimendan improves renal outcome in cardiac surgery: a randomised trial. J Cardiothorac Vasc Anesth.

[49] Balzer F, Treskatsch S, Spies C, Sander M, Kastrup M, Grubitzsch H (2014). Early administration of levosimendan is associated with improved kidney function after cardiac surgery, a retrospective analysis. J Cardiothorac Surg.

[50] Knezevic I, Poglajen G, Hrovat E, Oman A, Pintar T (2014). The effects of levosimendan on renal function early after heart transplantation: results from a pilot randomized trial. Clin Transplant.

[51] Fedele F (2011). Cost-effectiveness of levosimendan in patients with acute heart failure. J Cardiovasc Pharmacol.

